# Three-State Dielectric Switching within a Narrow Temperature Range in Isopropylammonium Lead Iodide, a One-Dimensional Perovskite with Polar Phase

**DOI:** 10.1021/acsami.4c03413

**Published:** 2024-05-22

**Authors:** Katarzyna Fedoruk-Piskorska, Jan K. Zaręba, Szymon J. Zelewski, Anna Gągor, Mirosław Mączka, Sławomir Drobczyński, Adam Sieradzki

**Affiliations:** † Department of Experimental Physics, Wrocław University of Science and Technology, Wybrzeże Wyspiańskiego 27, 50-370 Wrocław, Poland; ‡ Institute of Advanced Materials, Faculty of Chemistry, Wrocław University of Science and Technology, Wybrzeże Wyspiańskiego 27, 50-370 Wrocław, Poland; § W. Trzebiatowski Institute of Low Temperature and Structure Research, Polish Academy of Sciences, Okólna 2, 50-422 Wroclaw, Poland; ∥ Department of Optics and Photonics, Wrocław University of Science and Technology, Wybrzeże Wyspiańskiego 27, 50-370 Wrocław, Poland

**Keywords:** dielectric, switching, nonbinary, three-state, phase, ISOPrPbI_3_

## Abstract

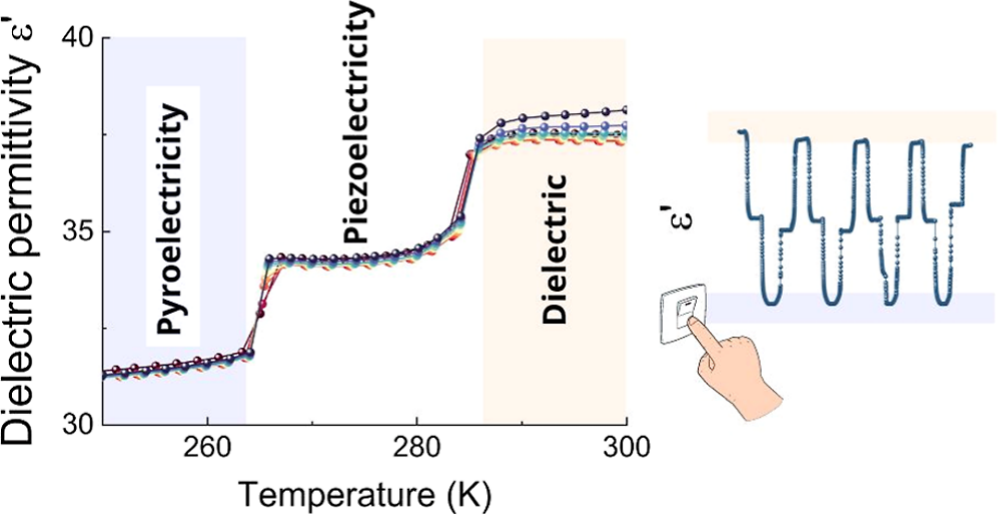

The phenomenon of dielectric switching has garnered considerable attention due to its potential applications in electronic and photonic devices. Typically, hybrid organic–inorganic perovskites, HOIPs, exhibit a binary (low–high) dielectric state transition, which, while useful, represents only the tip of the iceberg in terms of functional relevance. One way to boost the versatility of applications is the discovery of materials capable of nonbinary switching schemes, such as three-state dielectric switching. The ideal candidate for that task would exhibit a trio of attributes: two reversible, first-order phase transitions across three distinct crystal phases, minimal thermal hysteresis, and pronounced, step-like variations in dielectric permittivity, with a substantial change in its real part. Here, we demonstrate a one-dimensional lead halide perovskite with the formula (CH_3_)_2_C(H)NH_3_)PbI_3_, abbreviated as ISOPrPbI_3_, that fulfills these criteria and demonstrates three-state dielectric switching within a narrow temperature range of ca. 45 K. Studies on ISOPrPbI_3_ also revealed the polar nature of the low-temperature phase III below 266 K through pyrocurrent experiments, and the noncentrosymmetric character of the intermediate phase II and low-temperature phase III is confirmed via second harmonic generation measurements. Additionally, luminescence studies of ISOPrPbI_3_ have demonstrated combined broadband and narrow emission properties. The introduction of ISOPrPbI_3_ as a three-state dielectric switch not only addresses the limitations posed by the wide thermal gap between dielectric states in previous materials but also opens new avenues for the development of nonbinary dielectric switchable materials.

## Introduction

Hybrid organic–inorganic perovskites (HOIPs) have attracted considerable attention owing to their distinctive optoelectronic properties, particularly in photovoltaic applications.^1^ In the past decade, the greatest publicity has been given to the three-dimensional (3D) structures, with the general formula ABX_3_, where A represents an organic cation (currently limited to four cations), B is a metal ion, and X denotes a halide anion.^2^ However, the exploration of low-dimensional structures, such as two-dimensional (2D), one-dimensional (1D), and zero-dimensional perovskites, has emerged as a promising strategy to overcome the constraints of known 3D HOIPs.^3^ Indeed, 2D perovskites exhibit improved chemical and mechanical stability, coupled with diminished susceptibility to deleterious humidity factors.^4,5^ Additionally, the precise control of bandgap through tuning the thickness of organic and inorganic layers, as well as a wide selection of organic cations, is pivotal for various optoelectronic applications.^6^ In 2D structures, reduced self-trapping of charge carriers enhances transport and minimizes recombination losses In 2D structures, reduced self-trapping of charge carriers enhances transport and minimizes recombination losses. Moreover, these effects are additionally modulated by quantum and dielectric confinement, impacting electronic and optical properties.^7−9^ Further reduction of perovskite dimensionality to 1D is associated with the preferred edge-sharing coordination between lead halide PbX_6_ octahedra, affording elongated chain or wire-like arrangements, surrounded by organic cations. 1D structures, in turn, are anticipated to exhibit pronounced exciton–phonon coupling, owing to the distortion of the lead-halide coordination chain structure upon photoexcitation. This kind of coupling enhances the exciton self-trapping, typically leading to very broad emissions that can be harnessed for applications such as white-light emission.^10−12^

Dielectric confinement, influencing the behavior of free carriers, remains one of the critical parameters across different dimensionalities.^13^ Recently, HOIP materials have also become relevant in the context of dielectric switching due to their structural and chemical diversity. Dielectric switching refers to the phenomenon where the dielectric properties of a material sharply change in response to an external stimulus, such as an electric field, temperature, and pressure changes.^14^ It takes place in some materials undergoing structural first-order phase transitions (PTs), leveraging differences in dielectric permittivity between neighboring crystal phases. Accordingly, controlling the dielectric permittivity through the selection of organic and inorganic components of HOIP compounds is vital.^14−17^ While the two-state switching between low and high dielectric states is a fairly common phenomenon for HOIPs in general, in the case of 1D perovskite lead iodide materials there are only a handful of instances showing this specific electric functionality.^16,18−20^ A three-state dielectric switching is much more rare and material-demanding property, as it necessitates the presence of three structural phases within a reasonably narrow temperature range. Ideally, these phases should possess several key attributes: (i) reversibly interconvert between each other through a discontinuous PTs mechanism, (ii) feature low thermal hysteresis, and (iii) display step-like changes in the dielectric permittivity value, preferably with a high difference in ε′. A literature search indicates that so far only three compounds with formulas of [(CH_3_)_3_NOH][CdCl_3_], [CPtmp][Cd(SCN)_3_], and [(CH_3_)_3_PCH_2_F]CdCl_2_Br come close to fulfilling these requirements. Nevertheless, only for the latter the dielectric switching between three distinct states is demonstrated through the temperature cycling experiment, yet a significant drawback constitutes a huge thermal distance between the low and high dielectric states (Δ*T* of ca. 90 K), due to the broad temperature stability range of the intermediate phase.^21−23^ Therefore, the construction of the three-state dielectric switch that embodies all the attributes necessary for applications is a goal still to be achieved.

In this article, we demonstrate a lead halide 1D perovskite of formula (CH_3_)_2_C(H)NH_3_)PbI_3_ (ISOPrPbI_3_, where ISO stands for isopropylammonium cation) that represents the first example of fully functional HOIP three-state dielectric switch: it features three crystal phases within a narrow temperature range close to room temperature (RT), the first-order transition behavior of two reversible PTs, and shows high-contrast step-like changes in the dielectric permittivity that allow clear differentiation between low, intermediate and high states for phases I, II, and III, respectively. Our findings also reveal the polar character of the low-temperature (LT) phase III through the pyrocurrent experiments, and the confirmed noncentrosymmetricity via second harmonic generation (SHG) in the intermediate phase II. Luminescence measurements of ISOPrPbI_3_ also reveal broadband emission properties.

## Experimental Details

### Synthesis

Single crystals of ISOPrPbI_3_ were grown using a slow hydrolysis method, which proved successful for growing large single crystals of lead iodide perovskites.^24,25^ In this method, 4 mmol of PbI_2_ and 4 mmol of isopropylamine (99.5%, Sigma-Aldrich) were dissolved in a mixture of propylene carbonate (PC, 99.7%, Sigma-Aldrich) and HI (57 wt % in H_2_O, stabilized with H_3_PO_2_, Sigma-Aldrich) under stirring on a hot plate (50 °C). The PC/HI volume ratio was 2.6:1. The clear solution was transferred into a glass vial, which was kept at 50 °C for 2–3 days. The yellow crystals with dimensions up to 4 mm, which grew on the bottom of the vial, were separated from the liquid and dried at RT. A good match of the experimental powder X-ray diffraction pattern with the calculated one based on the single-crystal data (Figure S1) proved the phase purity of the bulk samples.

### Powder X-ray Diffraction

Powder X-ray diffraction of the ground crystals was measured in the reflection mode using an X’Pert PRO X-ray diffraction system equipped with a PIXcel ultrafast line detector and Soller slits for Cu Kα radiation (λ = 1.54056 Å).

### Differential Scanning Calorimetry

Differential scanning calorimetry (DSC) was performed using a Mettler Toledo DSC-3 calorimeter in the nitrogen atmosphere. The heating/cooling rate of 5 K min^–1^ was used in the temperature range of 130–400 K. The mass of the measured sample was 42.60 mg. The excess heat capacity associated with the PT was calculated by subtracting from the data the baseline representing the system variation in the absence of the PTs.

### Single-Crystal X-ray Diffraction

The RT (phase I, 293 K) and LT (phase III, 150 K) single-crystal X-ray diffraction data were collected on an Xcalibur diffractometer equipped with a 2D Atlas detector and Mo *K*α radiation source. Absorption was corrected using multiscan methods and spherical harmonics implemented in a SCALE3 ABSPACK scaling algorithm in *CrysAlis PRO* 1.171.38.41 (Rigaku Oxford Diffraction, 2015). The crystal structures were solved at 150 and 293 K by direct methods in SHELXT 2018/2^26^ and refined using SHELXL^27^ and Olex2 1.5.^28^ Octahedral distortion parameters were calculated in Vesta.^29^ H atoms were constrained during the refinement. The details concerning the crystal, data collection, and refinement results are shown in Table S1 in Supporting Information, and selected bonds and angles in Table S2. Hydrogen bond parameters at 150 K are collected in Table S3. The brief structure details are as follows:

((CH_3_)_2_C(H)NH_3_)[PbI_3_],·(phase I, *T* = 293 K): orthorhombic, *Cmcm*, *a* = 8.846 (2) Å, *b* = 17.180(2) Å, *c* = 8.0466(6) Å, *V* = 1222.9 (3) Å^3^, Z = 4, *R*[*F*^2^ > 2σ(*F*^2^)] = 0.041, w*R*(*F*^2^) = 0.110; (LT, *T* = 150 K): monoclinic, *P*2_1_, *a* = 10.2569(9) Å, *b* = 8.0650(6) Å, *c* = 14.055(2) Å, β = 90.03°, *V* = 1162.6 (2) Å^3^, *Z* = 4; *R*[*F*^2^ > 2σ(*F*^2^)] = 0.051, w*R*(*F*^2^) = 0.147, refined as two component twin. Despite numerous attempts, we were not able to determine the structure of the intermediate phase, as it was already twinned with significant peak overlap.

### Electrical Measurements

Dielectric measurements were carried out using a Broadband Impedance Novocontrol Alpha-A analyzer. Dielectric measurement was performed on a single-crystal sample with a surface area of 3.6 mm^2^ and a thickness of 0.99 mm. The silver paste was deposited on the crystal surface to ensure good electrical contact. The measurement was made in the temperature range from 150 to 350 K. The Novocontrol Quattro system was employed to stabilize the temperature, with nitrogen serving as the coolant. A pyroelectric current measurement was performed on a single crystal with silver electrical contacts. The sample was gradually cooled to a temperature of 235 K by subjecting it to an electric field of 250 V/mm. Current measurements were carried out using a Keithley 6514 electrometer during the heating of the sample from 235 to 300 K, with a controlled heating rate of 2 K/min. The polarization vs electric field measurement was performed using a Precision Premier II Ferroelectric tester. The conductive silver paste electrodes were applied to a single crystal with an area of 5 × 10^–2^ cm^2^ and a thickness of 700 μm. A maximum voltage of 400 V and a frequency of 1 Hz were applied across the sample, and data were recorded using the Virtual software. The cooling gas was nitrogen, and the measurement was carried out at a temperature of 250 K.

### Nonlinear Optical Studies

Nonlinear optical experiments were performed using a laser system employing a wavelength-tunable Topaz Prime Vis-NIR optical parametric amplifier (OPA) pumped by a Coherent Astrella Ti/sapphire regenerative amplifier providing femtosecond laser pulses (800 nm, 75 fs) at 1 kHz repetition rate. The output of OPA was set to 1400 nm, and the laser fluence at samples was equal to 0.19 mJ/cm^2^. The single crystals of ISOPrPbI_3_ and KDP were crushed with a spatula and sieved through an Aldrich mini-sieve set, collecting a microcrystal size fraction of 88–125 μm. Next, size-graded samples were fixed in between microscope glass slides to form tightly packed layers, sealed, and mounted to the horizontally aligned sample holder. No refractive index matching oil was used. The employed measurement setup operates in the reflection mode. Specifically, the laser beam was directed onto the sample at 45 deg to its surface. Emission collecting optics consisted of a Ø25.0 mm plano-convex lens of focal length 25.4 mm mounted to the 400 μm 0.22 NA glass optical fiber and was placed along the normal to the sample surface. The distance between the collection lens, and the sample was equal to 30 mm. The spectra of the temperature-dependent NLO responses were recorded by an Ocean Optics Flame T XR fiber-coupled CCD spectrograph with a 200 μm entrance slit. Scattered pumping radiation was suppressed with the use of a Thorlabs 750 nm hard-coated short-pass dielectric filter. The temperature control of the sample was performed using a Linkam LTS420 heating/freezing stage. Temperature stability was equal to 0.1 K. TR-SHG study of ISOPrPbI_3_ was conducted in a range of 223–323 K. Kurtz–Perry powder test was performed by comparing the SHG signal of ISOPrPbI_3_ at 273 K vs that of the KDP standard at 293 K.

### Linear Optical Studies

All photoluminescence (PL) measurements were taken using a custom-built microscopic system working in the epi-illumination configuration. The samples were excited with a 405 nm continuous wave diode laser beam focused through a 4×, NA = 0.10 microscope objective, also used for collecting the emitted light. The laser spot size (1/e^2^ diameter) was determined by imaging a microscope calibration slide at the focal plane. A multiline thermoelectrically cooled CCD detector (Avantes HSC1024 × 58TEC-EVO) was used for analyzing the PL spectra. To obtain the temperature dependence of PL, the crystal samples were attached to a sapphire plate with silver paste, and further mounted in a Linkam FTIR600 liquid-nitrogen-cooled microscopic stage, offering temperature control with stability better than 0.1 K and maintaining a heating/cooling rate of 5 K/min.

## Results

### Thermal Properties

**Figure 1:**
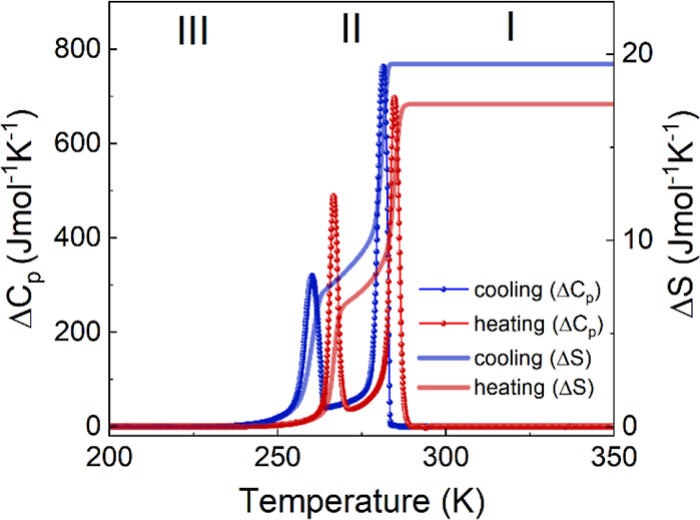
Changes in heat capacity (Δ*C*_p_) and entropy (Δ*S*) related to PTs in the heating (red) and cooling (blue) runs.

The DSC measurement showed two reversible PTs at *T*_1_ = 284.7 K (281.4 K) and *T*_2_ = 266.7 K (260.1 K) visible in the heating (cooling) mode (Figure S2). The presence of symmetrical, distinct peaks observed in changes in heat capacity (Δ*C*_p_) as a function of temperature, along with the noticeable thermal hysteresis observed between heating and cooling cycles, indicative of first-order PTs (Figure 1). This is also confirmed by the accompanying discontinuous change in the entropy (Δ*S*) (Figure 1). The change in entropy Δ*S* was estimated to be 6.5 J mol^–1^ K^–1^ (7.3 J mol^–1^ K^–1^) during the PT from phase III to II and 10.8 J mol^–1^ K^–1^ (12.2 J mol^–1^ K^–1^) from phase II to I in heating (cooling) mode. Based on the Boltzmann equation, Δ*S* = *R*ln(N), where Δ*S* is average entropy change, *R* is the gas constant, and *N* denotes the ratio of the number of distinguishable orientations, the values of *N* equal to approximately 2 (from phase III to II) and 4 (from phase II to I) was obtained. This result shows that greater changes occur during the transition from the centrosymmetric phase (I) to the noncentrosymmetric phase (II) than from the noncentrosymmetric phase (II) to the polar phase (III), which is confirmed by the results.

### Crystal Structure

**Figure 2:**
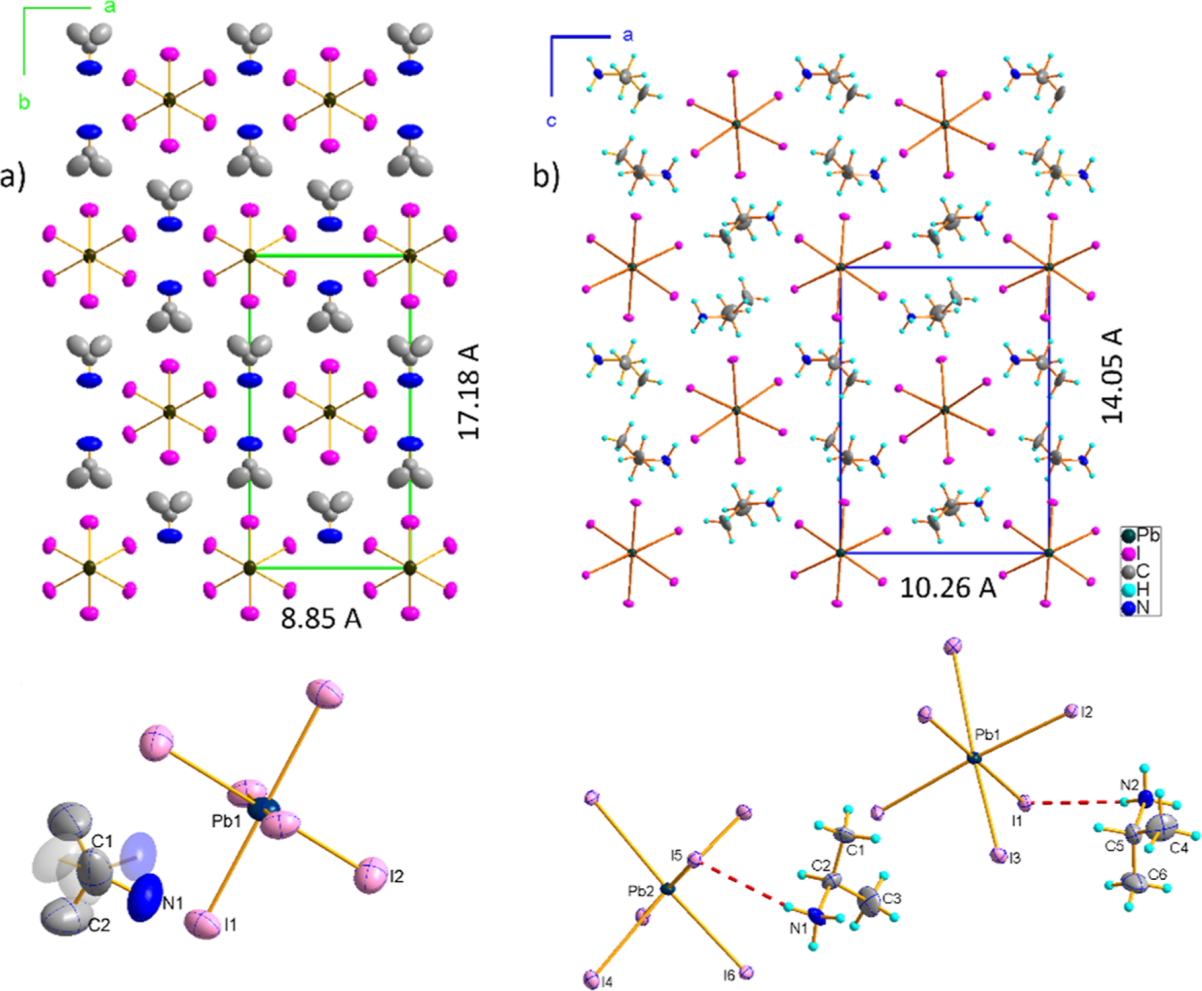
Crystal packing of ISOPrPbI_3_ shows the pseudohexagonal crystal structure, (a) orthorhombic phase I, *T* = 293 K, (b) monoclinic phase III, *T* = 150 K; the pictures at the bottom present basic building units in both phases, inequivalent atoms are labeled, displacement parameters are given at 50% probability label.

**Figure 3:**
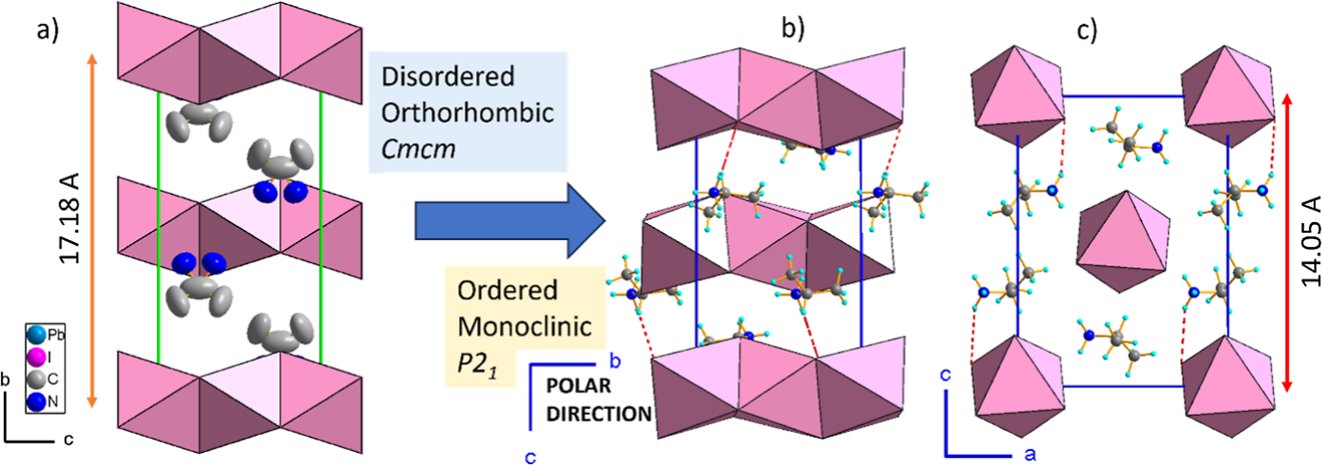
Crystal structure of ISOPrPbI_3_ (a) in the orthorhombic *Cmcm* phase I with disordered ISOPr^+^ cations, (b) packing in the monoclinic phase III, with ordered ISOPr^+^ cations interacting via N–H···I hydrogen bonds with the inorganic chains (dashed red lines), and (c) view of the structure in the [010] direction.

The crystal structure of ISOPrPbI_3_ aligns with the structural patterns observed in a variety of low-dimensional perovskites of APbI_3_ stoichiometry, where A stands for protonated amine. This structural family includes well-studied phases, such as the yellow δ-phase of FAPbI_3_^30^ and MAPbI_3_,^31^ as well as other related structures such as PyPbI_3_,^32^ DEAECPbI_3_,^33^ or other CsNiBr_3_-type compounds.^34^ The ISOPrPbI_3_ crystal structure exhibits 1D anionic chains constructed from face-sharing [PbI_6_]^4–^ octahedra. Organic counterions are distributed around the chains, and together with the complex anions form a pseudohexagonal assembly. Phase I of ISOPrPbI_3_ (I) is orthorhombic, of *Cmcm* symmetry, and is characterized by disordered ISOPr^+^ cations, which may adopt at least two symmetry equivalent sites due to the *m*2*m* symmetry of the central carbon atom. Figures 2a and 3a illustrate the crystal packing and basic structural units of this phase. The coordination sphere of lead exhibits *C*_2*h*_ site symmetry, with Pb–I distance showing little variation ranging between 3.2212(14) and 3.2340(12) Å. Iodine ions are ordered and form an almost ideal staggered conformation. Large displacement parameters of the inorganic moiety as well as the cations disorder imply thermally induced disorder of this phase.

The phase III structure of ISOPrPbI_3_ is polar and monoclinic, belonging to the *P*2_1_ space group. The monoclinic distortion is minimal, with the β angle slightly deviating to 90.03(1)°. The direct evidence of symmetry reduction from the orthorhombic to monoclinic system is the ferroelastic domain structure observed in phase III. This PT is characterized by the ordering of ISOPr^+^ cations and significant reorientation of the molecular part, facilitated by the formation of N–H···I hydrogen bonds.

It is noteworthy that, in phase III, all hydrogen atoms from NH_3_ groups are involved in hydrogen bonding, with donor-to-acceptor distances N···I in a narrow range between 3.69(3) and 3.79(3) Å. The rearrangement of the organic substructure contributes to reduced symmetry within the chains as well as notable alterations in interchain distances. The most pronounced changes are observed in the plane perpendicular to the chains, as depicted in Figures 2b and 3b.

In the monoclinic [001] direction, the distance contracts by approximately 18%, from 17.180(2) Å down to 14.055(1) Å. Conversely, in the monoclinic [100] direction, it elongates from 8.846(2) to 10.257(1) Å, indicating negative linear thermal expansion in this direction, with an almost 14% change in length between RT and 150 K. Less significant alterations are observed in the polar [010] direction, increasing from 8.047(1) at phase I to 8.065(1) at 150 K, also indicating the negative thermal expansion.

The distortion extends to interatomic distances and angles within the chains. In phase III, there are two independent PbI_3_ units, each forming a symmetry-inequivalent chain. However, the differences between the chains are subtle. Both Pb(1) and Pb(2) atoms occupy C1 sites, with Pb(1)-I distances ranging between 3.096(3) and 3.334(3) Å, and Pb(2)-I between 3.096(3) and 3.329(3) Å. Bond length distortion (Δ) and angle variance (σ^2^) are the same for both Pb(1)I_6_ and Pb(2)I_6_ octahedra, equaling 0.02 and 34 deg.^2^, respectively. The same parameters in phase I are as follows: Δ = 0.0017, σ^2^ = 25 deg.^2^ denoting only minor distortion of the chains in the monoclinic phase. Figure S3 in Supporting Information illustrates the deformation of the face-sharing octahedra in both phases.

### Dielectric Spectroscopy and Nonlinear Electrical Measurements

**Figure 4:**
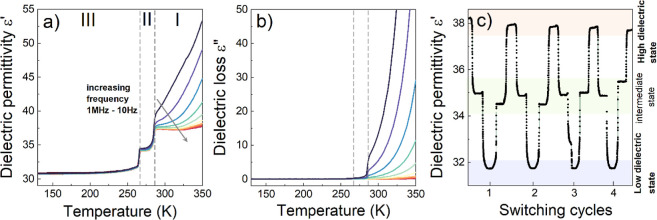
Temperature dependence of (a) real and (b) imaginary parts of the dielectric permittivity of the single crystal sample. (c) Several cycles of the temperature-induced dielectric switching of ISOPrPbI_3_ at a frequency of 0.5 MHz.

Broadband dielectric spectroscopy experiments were conducted to investigate the structural dynamics of ISOPrPbI_3_. The complex dielectric permittivity ε* (ε* = ε′ – iε″, where ε′ is the dielectric permittivity and ε″ is the dielectric loss) of the compound exhibits distinct changes at a temperature corresponding to the structural PTs (Figure 4a,b). A step-like anomaly of ε′ can be observed at approximately 265 and 285 K on cooling, which is according to the measurements and confirms the presence of first-order PTs (Figure 4a). During these changes ε′ values rise from 31.5 to 34.1 (III → II) and from 34.1 to 37.4 (II → I) for 1 MHz frequency. A step-like increase in the dielectric response associated with a first-order PT is a prerequisite for the functional property of dielectric switching.^19,35−39^ Since we identified two such transitions with abrupt changes of ε′ in a narrow temperature range (∼20 K), this prompted us to explore the possibility of dielectric switching at three temperature points. To this end, we probed ε′ changes by cyclically adjusting the temperature from 250 to 270 K and then to 295 K at a rate of 2 K/min. Following each cycle, a constant temperature (250, 270, and 295 K) was maintained for 5 min. Collected data (Figure 4c) unambiguously confirm the dielectric switching behavior involving three distinct dielectric states: a high dielectric state with ε′ at around 38, a low dielectric state close to ε′ = 32, complemented by an additional intermediate state with ε′ ∼ 35. Accordingly, ISOPrPbI_3_ can be seen as a unique example of the three-state dielectric switch, which operates in a very narrow temperature range. Performance-wise, one notes that ε′ remains stable and consistent throughout each cycle in the case of low and high dielectric states. This is not exactly the case for the intermediate dielectric state, i.e., the ε′ values are somewhat scattered from cycle to cycle with a standard deviation of 0.4. From the application point of view, such a relatively small variation of ε′ in the intermediate state should not be problematic since, in practice, the switching logic states are always defined for ranges rather than discrete values of physical properties. However, from a fundamental point of view, this behavior is intriguing. One tentative explanation is related to the fact that the medium state is probed at 270 K, which is very close to two adjacent crystal phases. DSC plot (Figure 1) shows that at that temperature point, the Δ*C*_p_ is nonzero and, hence, the value of ε′ that settles at that temperature is likely metastable. In a broader perspective, this result also suggests that the design of multistate dielectric switches working in tight temperature ranges may involve a trade-off in properties: the lower the separation of temperature points that define dielectric switch states, the lower the stability of ε′ value may be obtained.

We have also attempted to assess the contribution of conductivity to the obtained dielectric results. Indeed, above the temperature of 285 K, frequency dispersion for ε* is noticed, which is probably related to the presence of ionic/electron conductivity. In order to mitigate the influence of the electrode’s conductivity component at high temperatures, the modulus representation (M* = 1/ε*) was employed (Figure S4). The characteristic shapes of modulus curves, such as step shape (*M*′) and bell shape (*M*″), corroborate the occurrence of the conductivity process at high temperatures.

**Figure 5:**
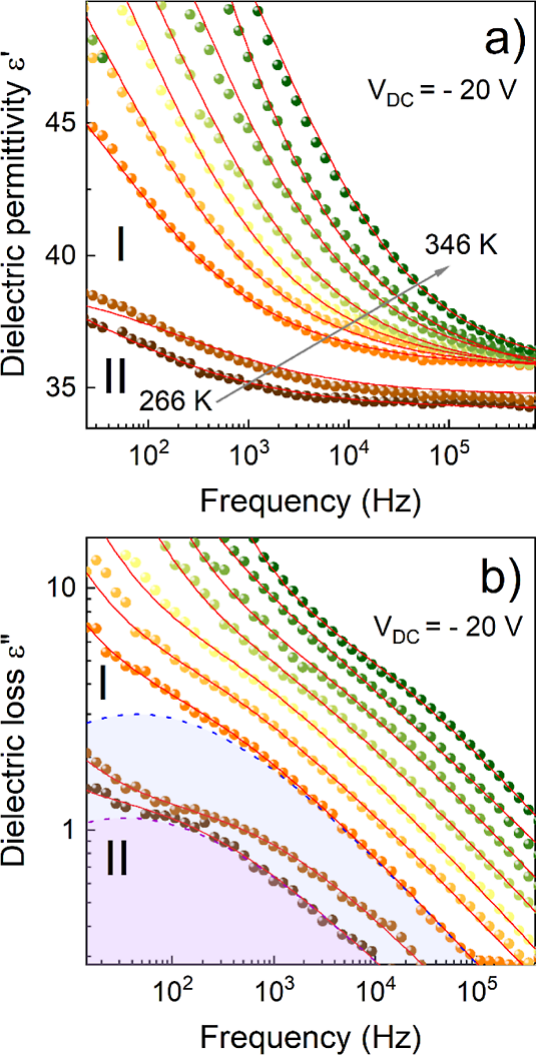
Curves of (a) dielectric permittivity and (b) dielectric losses as a function of frequency, with fitted Cole–Cole functions, obtained by measurement under an external DC electric field.

Dielectric studies under a constant direct current (DC) electric field further explain the structural dynamics of the material. The dielectric curves (ε′ and ε″) as a function of temperature, illustrated in Figure S5 for V_DC_= 20 V, match well with results obtained in the absence of an electric field (*V*_DC_ = 0), presented in Figure 4a,b. The primary distinction observed is the heightened frequency dispersion evident in phase II. However, notable variations emerged in the frequency domain, as depicted in Figures S4 and 5a,b. Measurements involving an external directional DC electric field revealed relaxation processes in both phases I and II (Figure 5a,b). To characterize the dipolar relaxation times, the data were fitted using the Cole–Cole function. Over the analyzed temperature range, the dependence of relaxation times exhibits a linear trend with the inverse temperature (1000/T) (Figure S6). Activation energies were estimated using the Arrhenius relationship (Figure S6) and are equal to 0.66 and 1.07 eV for phases I and II, respectively. This observed structural relaxation process is attributed to the reorientational movements of ISOPr^+^ cations in the I phase. The activation energy values align with those associated with the rotational movements of organic cations in HOIPs.^40^

**Figure 6:**
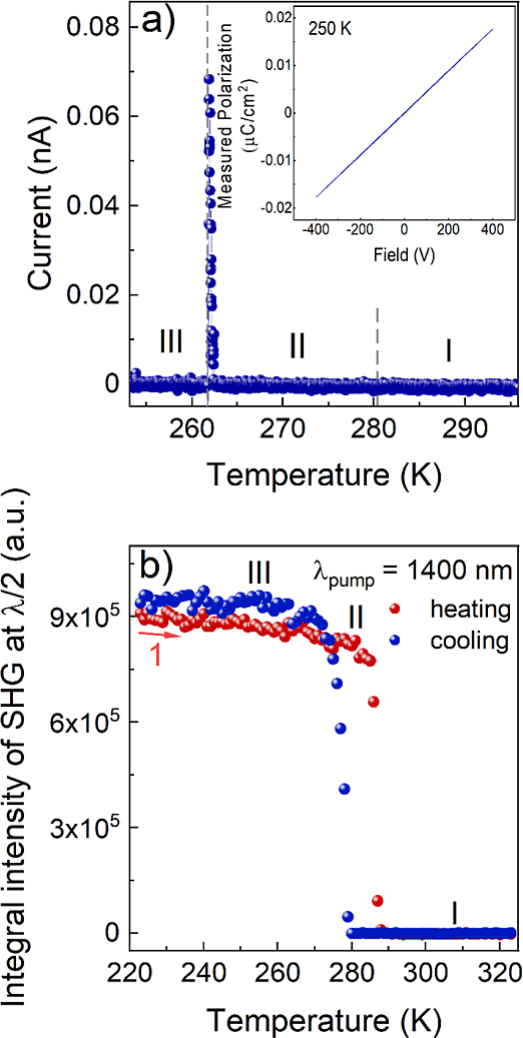
Temperature dependence of (a) the pyroelectric current of a single crystal after poling in a DC electric field is investigated, and (b) SHG integral intensities for ISOPrPbI_3_. The inset presents the measured polarization as a function of the applied electric field at a temperature of 250 K and a frequency of 1 Hz.

Pyroelectric current measured along the polar axis [010] as a function of temperature is presented in Figure 6a. The primary finding from the measurements is the pyroelectric current value at a temperature of approximately 260 K, corresponding to the transition from phase III to phase II. We did not observe any pyrocurrent at around 280 K when monitoring the transition from phase II to phase I. These results demonstrate that while phase III is polar, phase II cannot be polar; this does not preclude the possibility of structural noncentrosymmetry of intermediate phase II (see SHG studies section below). Furthermore, the linear relationship between polarization (*P*) and the applied electric field (*E*) confirms that phase III of ISOPrPbI_3_ material exhibits exclusively pyroelectric characteristics. A similar case was observed for the related compounds.^37,41^

### SHG Studies

The screening of the SHG activity in a wide temperature range (223–323 K) covering two structural PTs in ISOPrPbI_3_ was performed to spectroscopically confirm the noncentrosymmetric setting of the investigated crystal phases as well as to compare their relative SHG efficiency. Temperature plot of the integral intensities of SHG signals (λ_2ω_ = 700 nm), obtained upon irradiation with 1400 nm femtosecond laser pulses, is presented in Figure 6b, while experimental spectra are shown in Figures S7 and S8, Supporting Information. It is confirmed that phase I is centrosymmetric, as no SHG could be detected above 288 K (heating run) and 280 K (cooling run). However, below these temperature points, a clear SHG signal emerges, indicating an acentric setting of phase II. The presence of nearly 10 K-wide hysteresis supports the first-order character of the I → II PT. The SHG efficiency for phase II was estimated by employing a Kurtz–Perry test (Figure S9). The integral intensity of SHG for ISOPrPbI_3_ (at 273 K) is about 0.21 that of KDP (at 293 K) of the same particle size. Interestingly, an analysis of the temperature plot in the vicinity of PT II → III, which is at around 260 K, shows that this phase change has virtually no impact on the SHG intensity, despite the first-order mechanism inferred from DSC.

### PL Spectroscopy

**Figure 7:**
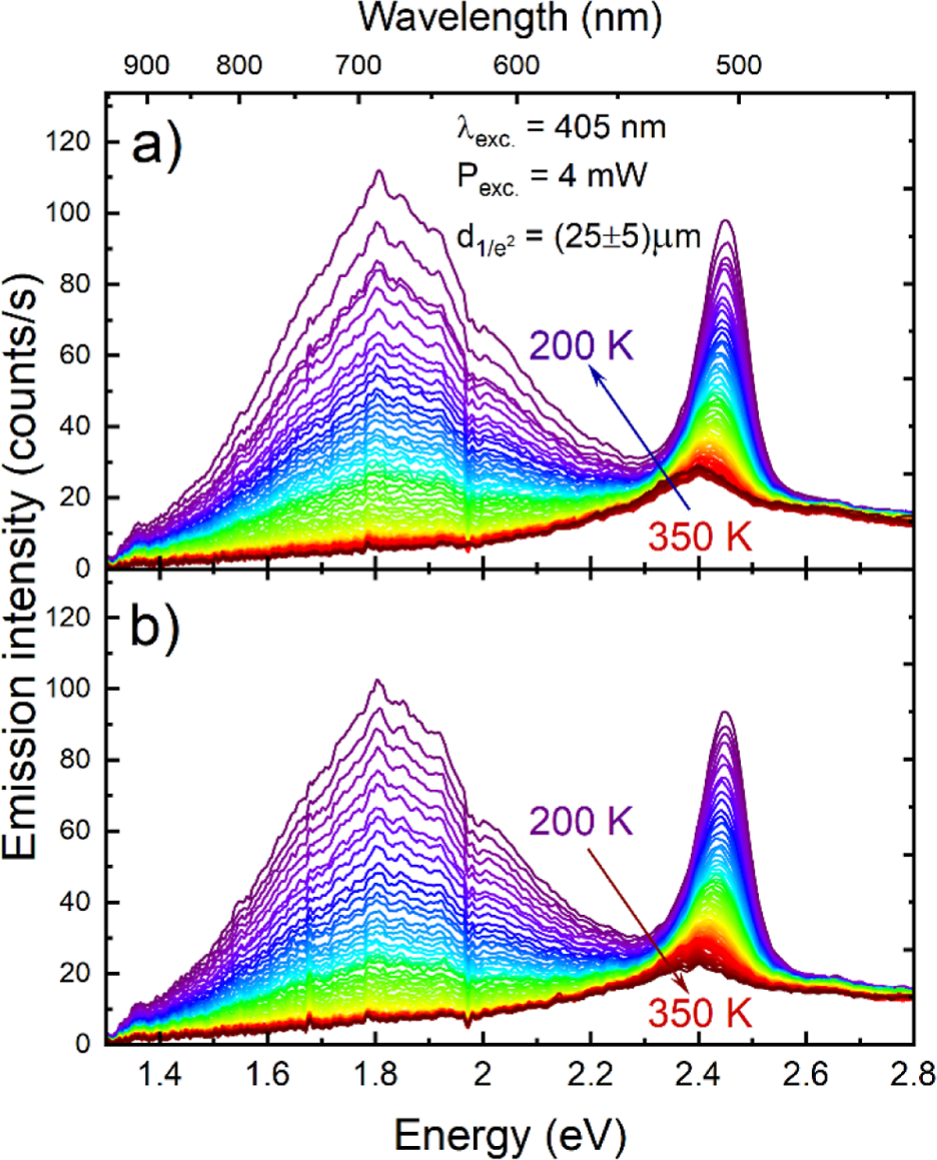
PL spectra acquired during (a) cooling and (b) heating cycles.

In search of further indications of PTs in ISOPrPbI_3_, we studied its optical properties by temperature-dependent PL spectroscopy. The experimental procedure was consistent with previous studies in terms of temperature cycling to enable quantitative comparison. At RT and above, up to 350 K at which the temperature scan started, the PL is dominated by a single peak around 2.4 eV (∼516 nm). Upon cooling down throughout the determined PTs temperatures (Figure 7a), another broad emission band centered at 1.8 eV (∼690 nm) emerges around 280 K. The intensity of both PL features monotonically increases while cooling the sample down to the base temperature of 200 K. The excitation laser power was optimized to obtain sufficient emission intensity even at the highest temperatures while avoiding photoinduced sample degradation and, hence, the preserved spectral shape during the heating cycle (Figure 7b).

**Figure 8:**
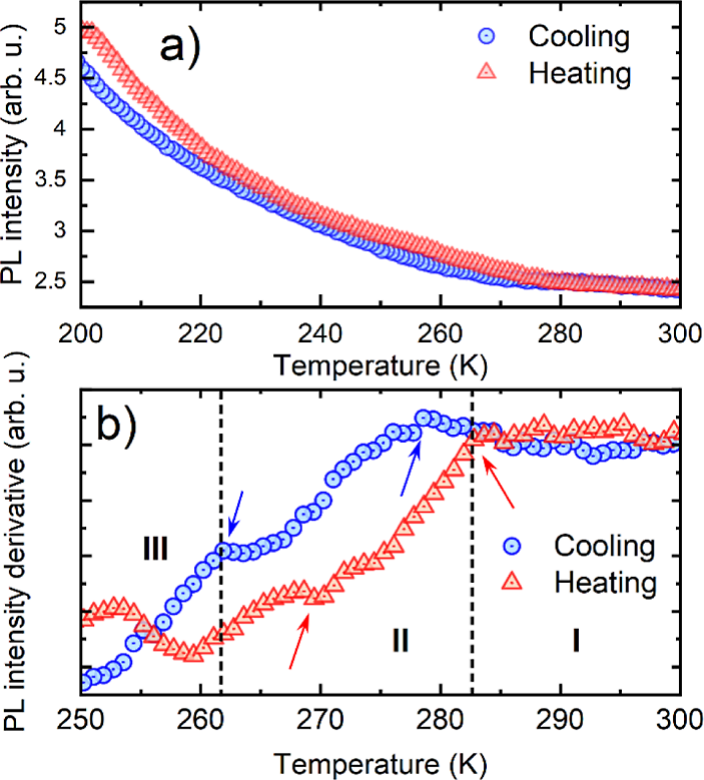
(a) Integrated emission intensity, (b) derivative analysis with denoted regions corresponding to the determined I–III phases. The arrows indicate the inflection points at the observed PT temperatures.

The integrated emission intensity, shown in Figure 8a, overall depicts monotonic quenching, showing minute changes in the temperature trends for both cooling and heating cycles. To enhance the visibility of possible subtle changes, we calculated the first derivative of the intensity traces (Figure 8b). This allows us to pinpoint deviations from the classic relation predicted by the Arrhenius formula caused by a combination of changes in, above all, the electron–phonon interaction strength and the dielectric environment and was demonstrated to be effective for supporting observations of PTs in our previous study.^20^ It is worth mentioning that the integrated intensity of the narrow peak constitutes 10–12% of the total emission intensity; hence, the analysis emphasizes changes in the broad, localized emission band. The derivative curves reveal a significant change in the intensity trend at 279 (283) K during cooling (heating) cycles, in good agreement with the PT between phases I and II. Additionally, a less pronounced but discernible inflection point can be observed in the 260–270 K region, with the estimated PT between phases II–III at 262 and 268 K for cooling and heating cycles (marked with arrows), respectively. The results clearly indicate that PT hysteresis directly affects the optical activity in the material, with potential underlying mechanisms related to different defect activity or changes in dielectric screening.

## Conclusions

Despite previous studies demonstrating temperature-induced bistable dielectric switching in various materials, achieving a three-state dielectric switch with closely spaced dielectric states remained a significant challenge. In this paper, we present isopropylammonum lead iodide, ISOPrPbI_3_, a 1D perovskite that displays reversible temperature-induced three-state dielectric switching between low (ε′ = 32 at 250 K), intermediate (ε′ of ca. 35 at 270 K) and high (ε′ = 38 at 295 K) dielectric states for phases I, II, and III, respectively. This marks the first observed instance of dielectric switching among three phases within a narrow temperature range in proximity to RT. We note that for the low and high dielectric states, we observe that ε′ value maintains a stable and consistent profile throughout each cycle. However, this level of stability is not entirely mirrored in the intermediate dielectric state, where ε′ values exhibit some variability from cycle to cycle, with a standard deviation of 0.4. However, from an application standpoint, such minor fluctuations in the intermediate state’s ε′ are unlikely to pose significant issues since, in practical scenarios, the logic states for switching are defined over ranges of physical properties rather than fixed values. Accordingly, the observed variability within the intermediate state falls within acceptable limits for the operational integrity of dielectric switches, though the phenomenon itself of ε′ instability warrants further fundamental studies.

The three-state dielectric switching is directly related to the phase change characteristics, which show two distinct first-order PTs at *T*_1_ = 285 K and *T*_2_ = 267 K during heating, as inferred from DSC. Crystallographic analysis reveals that at RT the material adopts a centrosymmetric orthorhombic structure with the space group *Cmcm*. In contrast, the LT phase III belongs to the space group *P*2_1_. The noncentrosymmetric character of the intermediate phase II and LT phase III has been unequivocally established through SHG studies. In the case of the LT phase, the pyroelectric measurements validate the polar characteristics of the material. Additionally, dielectric studies under a constant electric field indicate relaxation processes associated with the reorientational dynamics of the ISOPr^+^ cations. Luminescence studies of ISOPrPbI_3_ have shown that luminescence spectra, which encompass both broadband and narrow emission, are very weakly influenced by the PTs and, hence, are less telling than the other techniques employed. All in all, the introduction of ISOPrPbI_3_ as a three-state dielectric switch not only overcomes the limitations of previous materials, characterized by a wide thermal gap between dielectric states but also heralds new possibilities for the development of nonbinary dielectric switchable materials.

## Supporting Information Available

Crystallographic data of ISOPrPbI_3_ at 150 K (CIF)
Crystallographic data of ISOPrPbI_3_ at 293 K (CIF)
Experimental details, geometric and hydrogen-bond parameters, experimental and calculated powder X-ray diffraction patterns, DSC trace, deformation of the octahedra, dielectric spectra, temperature dependence of the relaxation times, and SHG traces (PDF)

## Supplementary Material

am4c03413_si_001.cif

am4c03413_si_002.cif
