# Supplementary material for: Three-State Dielectric Switching within a Narrow Temperature Range in Isopropylammonium Lead Iodide, a One-Dimensional Perovskite with Polar Phase
Source: ACS Appl Mater Interfaces. 2024 May 22;16(22):28829–37. doi: 10.1021/acsami.4c03413 (PMC11163392; doi:10.1021/acsami.4c03413)
Supplement: Supplementary file 3 — am4c03413_si_003.pdf [file am4c03413_si_003.pdf]

Supporting information

Three-State Dielectric Switching Within a Narrow Temperature Range in Isopropylammonium Lead Iodide, A One-Dimensional Perovskite with Polar Phase

Katarzyna Fedoruk-Piskorska,[a] Jan K. Zaręba,[b] Szymon J. Zelewski,[a] Anna Gągor,[c] Mirosław Mączka,[c] Sławomir Drobczyński[d] and Adam Sieradzki*[a]*

[a]Department of Experimental Physics, Wrocław University of Science and Technology, Wybrzeże Wyspiańskiego 27, 50-370 Wrocław, Poland

[b]Institute of Advanced Materials, Faculty of Chemistry, Wrocław University of Science and Technology, Wybrzeże Wyspiańskiego 27, 50- 370, Wrocław, Poland

[c]W. Trzebiatowski Institute of Low Temperature and Structure Research, Polish Academy of Sciences, Okólna 2, 50-422 Wroclaw, Poland

[d]Department of Optics and Photonics, Wrocław University of Science and Technology, Wybrzeże Wyspiańskiego 27, 50-370 Wrocław, Poland

e-mail: katarzyna.fedoruk-piskorska@pwr.edu.pl adam.sieradzki@pwr.edu.pl

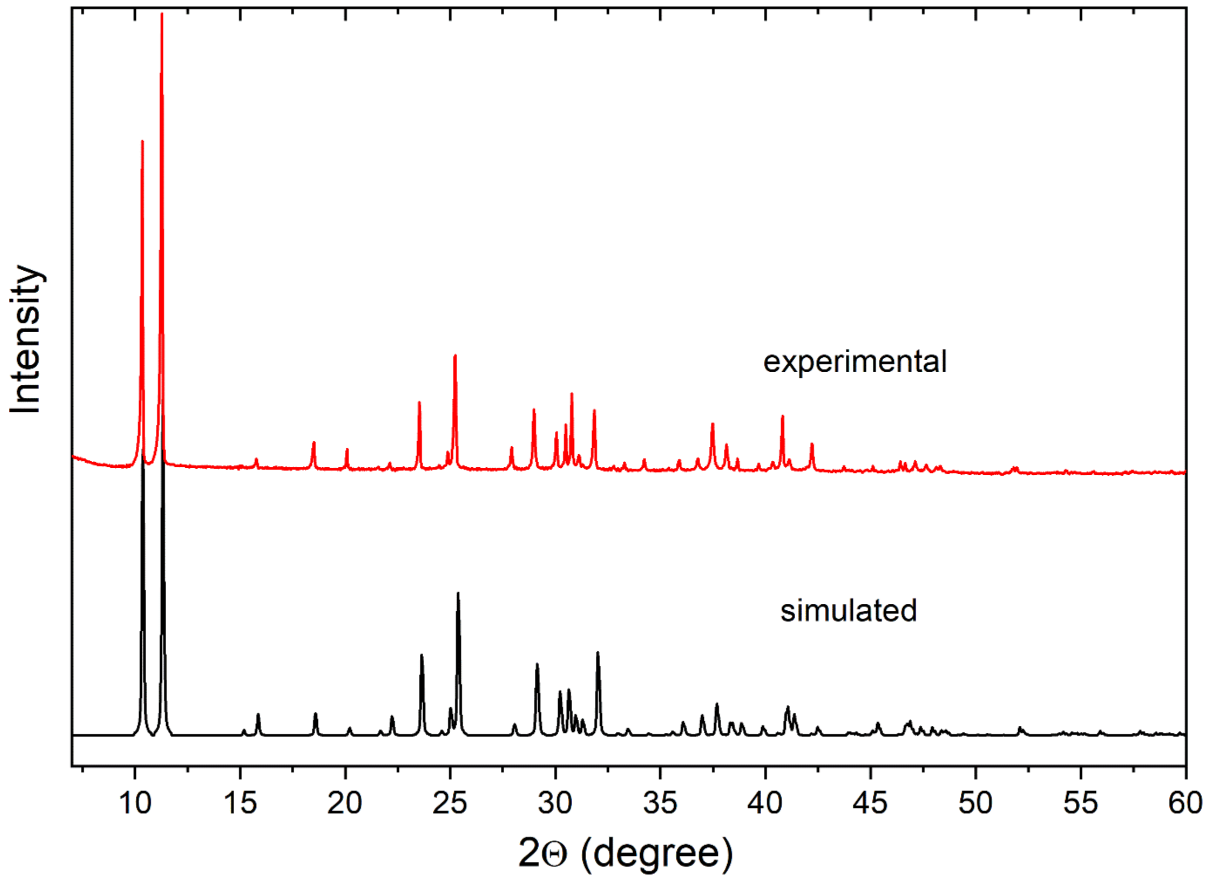

Figure S1. Experimental powder X-ray diffraction pattern of $ISOPrPbI_3$ together with the simulated pattern based on single-crystal room-temperature X-ray diffraction data.

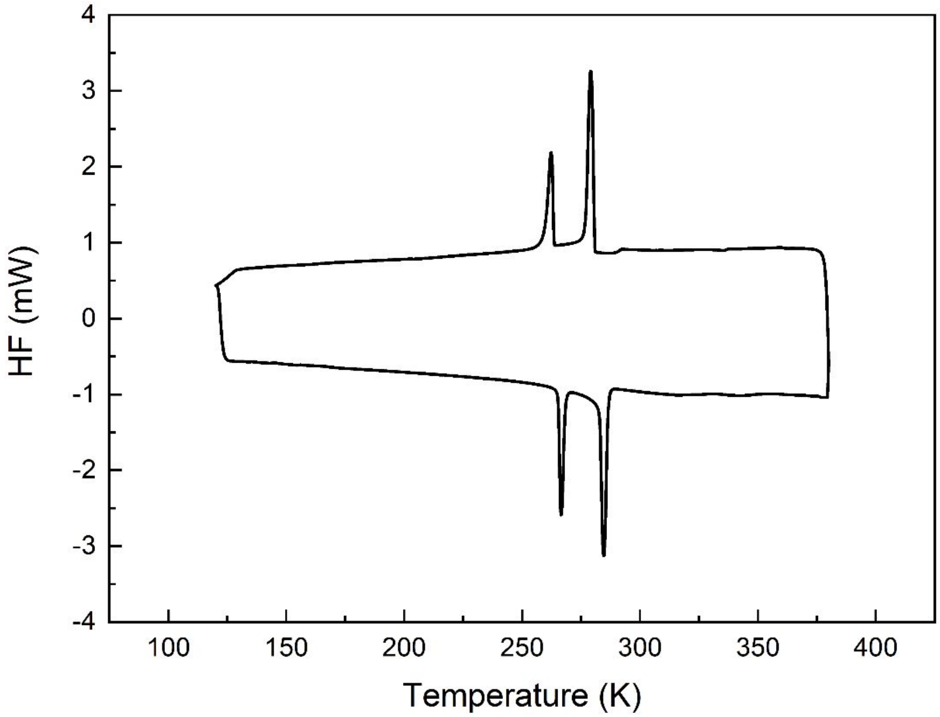

Figure S2. DSC trace of $ISOPrPbI_3$.

Table 1. Experimental details

For both structures: M_r = 648.01, Z = 4. Experiments were carried out with Mo *K*a radiation using a Xcalibur, Atlas. Absorption was corrected for by multi-scan methods, *CrysAlis PRO* 1.171.38.43 (Rigaku Oxford Diffraction, 2015) Empirical absorption correction using spherical harmonics, implemented in SCALE3 ABSPACK scaling algorithm.

	**PHASE I**	**PHASE III**
Crystal data		
Chemical formula	$I_3Pb \cdot C_3H_{10}N$	$I_3Pb\ C_3H_{10}N$
Crystal system, space group	Orthorhombic, *Cmcm*	Monoclinic, $P2_1$
Temperature (K)	292	150
a, *b*, *c* (Å)	8.846 (2), 17.180(2), 8.0466(6)	10.2569(9), 8.0650(6), 14.055(2)
a, b, γ (°)	90, 90, 90	90, 90.03(1), 90
V ($Å^3$)	1222.9 (3)	1162.64 (17)
μ (mm^{-1})	21.31	22.42
Crystal size (mm)	0.18 × 0.15 × 0.08	0.18 × 0.15 × 0.08
Data collection		

No. of measured, independent and observed [$I > 2\sigma(I)$] reflections	2139, 826, 435	9628, 9628, 7489
R_{int}	0.034	refined as two component twin
(sin q/l)$_{max}$ (Å^{-1})	0.686	0.695
Refinement		
$R[F^2 > 2s(F^2)]$, $wR(F^2)$, S	0.041, 0.110, 0.94	0.051, 0.147, 0.99
No. of reflections	826	9628
No. of parameters	34	152
No. of restraints	2	55
H-atom treatment	–	H-atom parameters constrained
Dρ_{max}, Dρ_{min} (e Å^{-3})	1.00, -0.73	2.09, -2.27
Absolute structure	–	Classical Flack method preferred over Parsons because s.u. lower.
Absolute structure parameter	–	0.449 (9)

Computer programs: *CrysAlis PRO* 1.171.38.43 (Rigaku OD, 2015), *SHELXT* 2018/2 (Sheldrick, 2018), *SHELXT* (Sheldrick, 2015), *SHELXL* 2018/3 (Sheldrick, 2015), Olex2 1.5 (Dolomanov *et al.*, 2009).

Table 2. Selected geometric parameters (Å, °)

PHASE I		PHASE III	
Pb1—I1^{i}	3.2340 (11)	Pb1—I1	3.244 (2)
Pb1—I1^{ii}	3.2340 (11)	Pb1—I1^{vi}	3.215 (2)
Pb1—I1^{iii}	3.2340 (11)	Pb1—I2^{vii}	3.264 (2)
Pb1—I1	3.2340 (11)	Pb1—I2	3.157 (2)
Pb1—I2^{i}	3.2212 (13)	Pb1—I3	3.096 (2)
Pb1—I2	3.2212 (13)	Pb1—I3^{vi}	3.334 (2)
		Pb2—I4	3.096 (2)
		Pb2—I4^{viii}	3.328 (2)
		Pb2—I6^{ix}	3.266 (3)
		Pb2—I6	3.152 (2)

		Pb2—I5	3.248 (2)
		Pb2—I5^{viii}	3.214 (2)
I1^{i}—Pb1—I1^{iii}	86.24 (4)	I1^{vi}—Pb1—I1	178.96 (6)
I1^{iii}—Pb1—I1	93.75 (4)	I1—Pb1—I2^{vii}	83.58 (6)
I1^{i}—Pb1—I1	180.0	I1^{vi}—Pb1—I2^{vii}	96.70 (6)
I1^{ii}—Pb1—I1	86.25 (4)	I1—Pb1—I3^{vi}	97.63 (6)
I1^{iii}—Pb1—I1^{ii}	180.00 (5)	I1^{vi}—Pb1—I3^{vi}	83.32 (6)
I1^{i}—Pb1—I1^{ii}	93.76 (4)	I2—Pb1—I1^{vi}	85.78 (6)
I2—Pb1—I1^{i}	84.82 (3)	I2—Pb1—I1	93.90 (6)
I2—Pb1—I1^{iii}	84.82 (3)	I2—Pb1—I2^{vii}	176.23 (8)
I2^{i}—Pb1—I1^{ii}	84.82 (3)	I2^{vii}—Pb1—I3^{vi}	99.05 (6)
I2^{i}—Pb1—I1^{iii}	95.18 (3)	I2—Pb1—I3^{vi}	84.04 (6)
I2^{i}—Pb1—I1^{i}	95.18 (3)	I3—Pb1—I1	86.72 (6)
I2^{i}—Pb1—I1	84.82 (3)	I3—Pb1—I1^{vi}	92.30 (6)
I2—Pb1—I1	95.18 (3)	I3—Pb1—I2	90.86 (7)
I2—Pb1—I1^{ii}	95.18 (3)	I3—Pb1—I2^{vii}	86.20 (6)
I2^{i}—Pb1—I2	180.0	I3—Pb1—I3^{vi}	173.50 (8)
Pb1—I1—Pb1^{iv}	76.93 (3)	I4—Pb2—I4^{viii}	173.61 (8)
Pb1—I2—Pb1^{v}	77.29 (4)	I4—Pb2—I6^{ix}	86.04 (6)
		I4—Pb2—I6	90.89 (7)
		I4—Pb2—I5	86.60 (6)
		I4—Pb2—I5^{viii}	92.31 (6)
		I6—Pb2—I4^{viii}	84.10 (6)

		$I6^{ix}$—Pb2—$I4^{viii}$	99.12 (6)
		I6—Pb2—$I6^{ix}$	176.07 (8)
		I6—Pb2—I5	93.87 (6)
		I6—Pb2—$I5^{viii}$	85.89 (6)
		$I5^{viii}$—Pb2—$I4^{viii}$	83.39 (6)
		I5—Pb2—$I4^{viii}$	97.67 (6)
		I5—Pb2—$I6^{ix}$	83.50 (6)
		$I5^{viii}$—Pb2—$I6^{ix}$	96.69 (7)
		$I5^{viii}$—Pb2—I5	178.89 (6)
		$Pb1^{vii}$—I1—Pb1	77.28 (4)
		Pb1—I2—$Pb1^{vi}$	77.80 (4)
		Pb2—I4—$Pb2^{ix}$	77.67 (4)
		Pb2—I6—$Pb2^{viii}$	77.84 (5)
		$Pb2^{ix}$—I5—Pb2	77.24 (4)
		Pb1—I3—$Pb1^{vii}$	77.59 (5)

Symmetry code(s): (i) -*x*+1, -*y*+1, -*z*+1; (ii) -*x*+1, *y*, -*z*+3/2; (iii) *x*, -*y*+1, *z*-1/2; (iv) -*x*+1, -*y*+1, *z*+1/2; (v) -*x*+1, -*y*+1, *z*-1/2; (vi) -*x*+1, *y*+1/2, -*z*+1; (vii) -*x*+1, *y*-1/2, -*z*+1; (viii) -*x*, *y*-1/2, -*z*; (ix) -*x*, *y*+1/2, -*z*.

Table 3. Selected hydrogen-bond parameters in PHASE III

D—H···*A*	*D*—H (Å)	H···*A* (Å)	*D*···*A* (Å)	*D*—H···*A* (°)
PHASE III				
N2—H2A···I1	0.91	2.89	3.71 (3)	149.3
N2—H2B···$I5^{i}$	0.91	2.89	3.78 (3)	167.2

N2—H2C···I4[ii]	0.91	3.03	3.74 (3)	135.6
N1—H1D···I3[iii]	0.91	2.93	3.69 (3)	141.2
N1—H1E···I5	0.91	2.88	3.72 (3)	154.6
N1—H1F···I1[iv]	0.91	2.92	3.79 (3)	159.8

Symmetry code(s): (i) *x*+1, *y*, *z*+1; (ii) -*x*+1, *y*+1/2, -*z*+1; (iii) -*x*, *y*-1/2, -*z*+1; (iv) *x*-1, *y*, *z*.

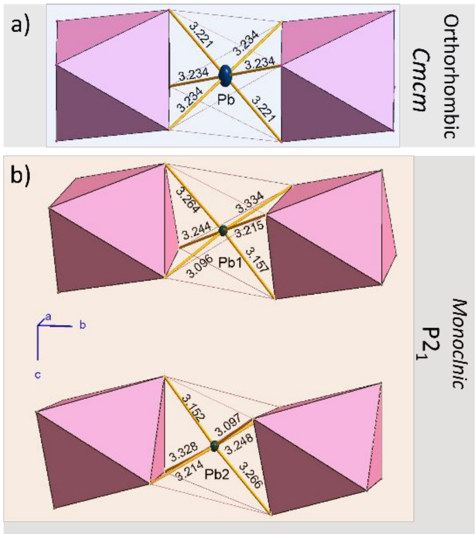

Figure S3. Deformation of the edge-shared octahedra in phases a) HT, orthorhombic, site symmetry of Pb ion C_{2h} b) LT, monoclinic, site symmetry of both Pb1 and Pb2 – C_1.

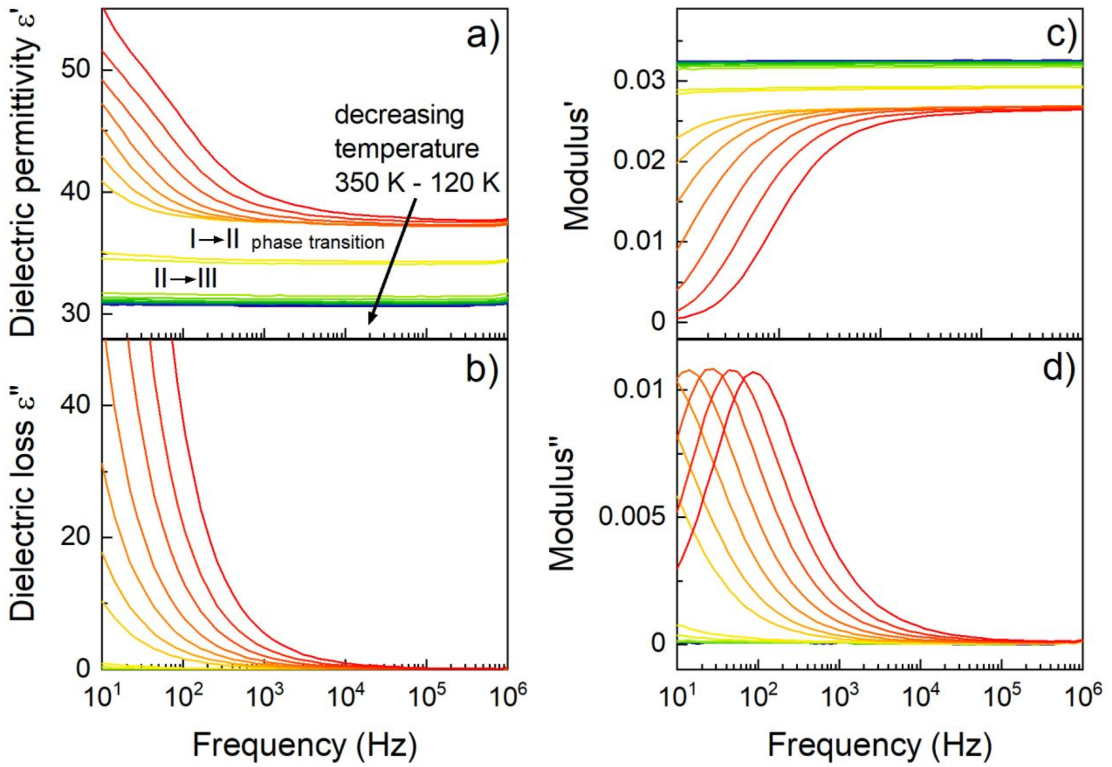

Figure S4. Frequency dependence of the a) dielectric permittivity, b) dielectric loss, c) real M′, and d) imaginary M″.

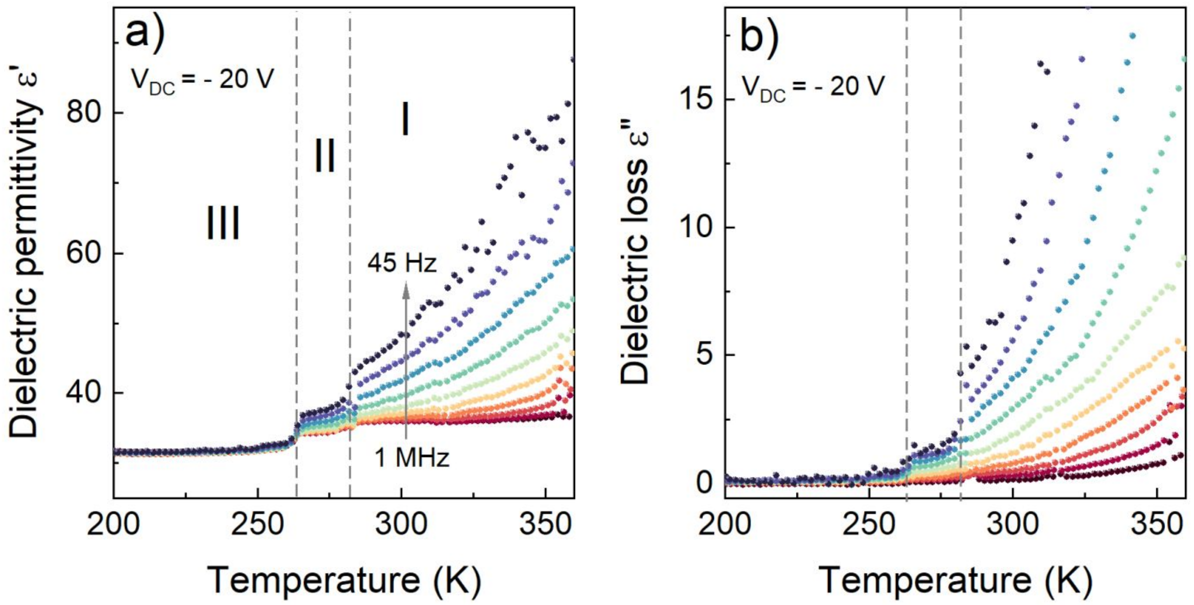

Figure S5. Temperature dependence of a) dielectric permittivity and b) dielectric losses of the $ISOPrPbI_3$ single crystal at an external electric field of -20 V.

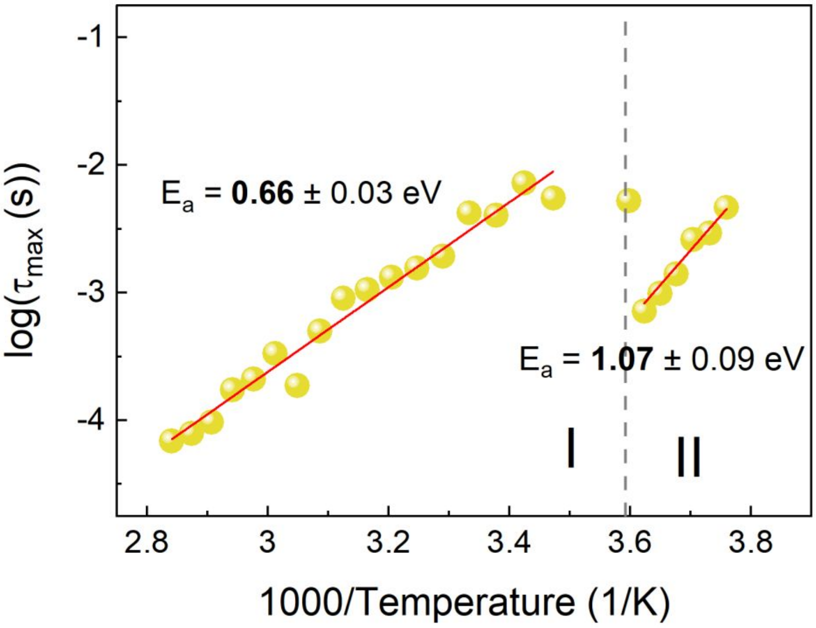

Figure S6. Inverse temperature (1000/T) dependence of relaxation times.

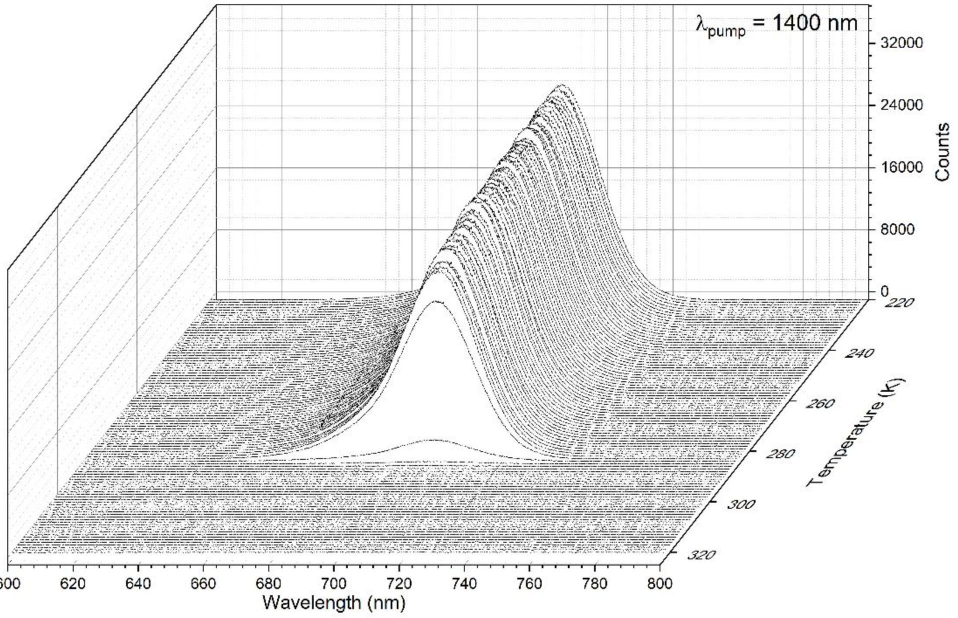

Figure S7. Overlay of experimental spectra obtained upon irradiation of $ISOPrPbI_3$ with 1400 nm femtosecond laser pulses during heating run from 223 K to 323 K.

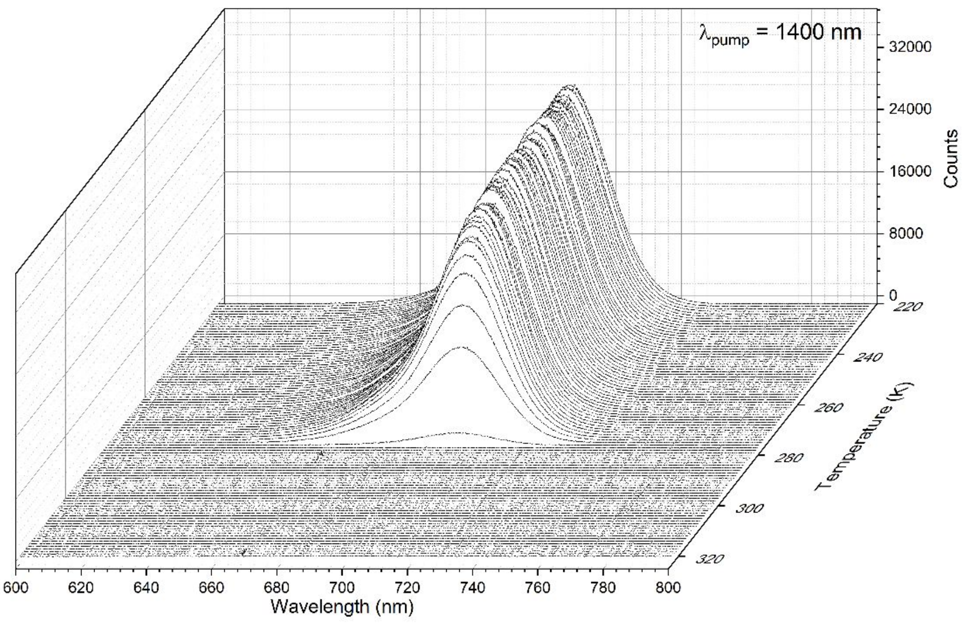

Figure S8. Overlay of experimental spectra obtained upon irradiation of $ISOPrPbI_3$ with 1400 nm femtosecond laser pulses during cooling run from 323 K to 223 K.

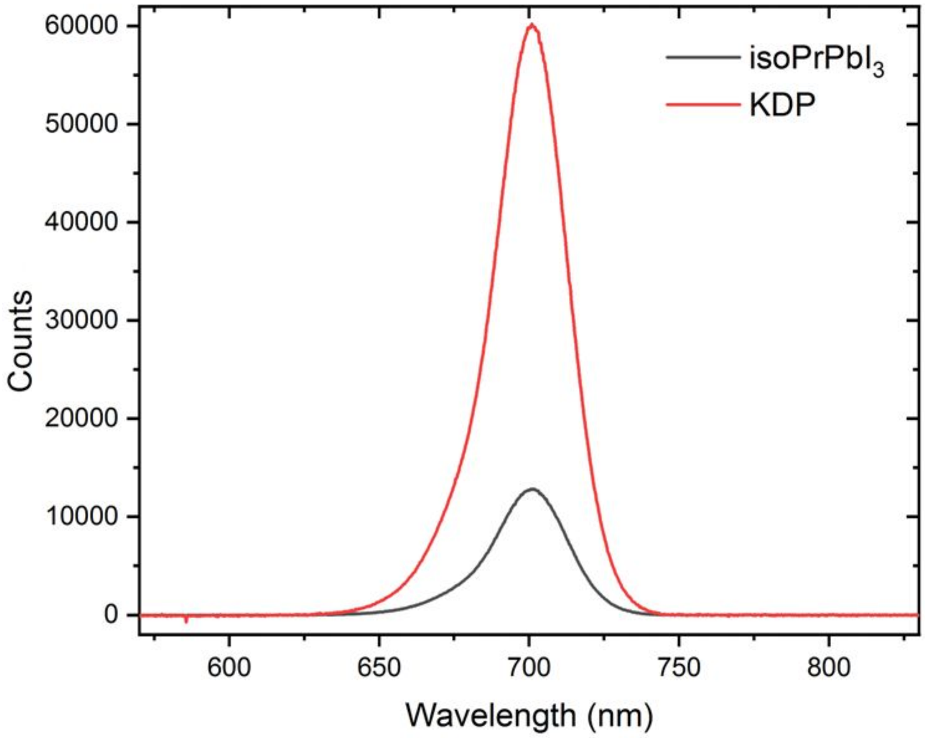

Figure S9. Overlay of SHG traces of $ISOPrPbI_3$ with that of KDP obtained upon irradiation with 1400 nm femtosecond laser pulses. $ISOPrPbI_3$ was cooled to 273 K, while KDP was measured at 293 K.
